# Objective quantification of ligament balancing using VERASENSE in measured resection and modified gap balance total knee arthroplasty

**DOI:** 10.1186/s12891-018-2190-8

**Published:** 2018-07-27

**Authors:** Kyu-Jin Cho, Jong-Keun Seon, Won-Young Jang, Chun-Gon Park, Eun-Kyoo Song

**Affiliations:** 1 Center for Joint Disease, Chonnam National University Hwasun Hospital, 160, Ilsim-Ri, Hwasun-Eup, Hwasun-Gun, Jeonnam 519-809 South Korea; 2 Department of Orthopaedic Surgery, Chonnam National University Hwasun Hospital, 160 Ilsim-Ri, Hwasun-gun, Jeonnam 519-809 South Korea

**Keywords:** Total knee arthroplasty, Orthosensor, Measured-resection, Gap-balance, Ligament balancing

## Abstract

**Background:**

Soft tissue balancing which is above all most important factor of total knee arthroplasty, has been performed by subjective methods. Recently objective orthosensor has been developed for compartment pressure measurement. The purpose of this study was: (1) to quantify the compartment pressure of the joint throughout the range of motion during TKA using orthosensor, (2) to determine the usefulness of orthosensor by analyzing correlation between the pressure in both compartment with initial trial and after final implantation, and (3) to evaluate the types and effectiveness of additional ligament balancing procedures to compartment pressure.

**Methods:**

Eighty-four patients underwent total knee arthroplasty (TKA) using VERASENSE Knee System. TKA was performed by measured resection and modified gap balance technique. Compartment pressure was recorded on full extension, 30°, 60°, 90° and full flexion at initial (INI), after each additional procedure, and after final (FIN) implantation. “Balanced” knees were defined as when the compartment pressure difference was less than 15 pounds.

**Results:**

Thirty patients (35.7%) showed balanced knee initially and 79 patients (94.0%) showed balance after final implantation. The proportion of balanced knee after initial bony resection, modified gap balancing TKAs showed significantly higher proportion than measured resection TKAs (*P* = 0.004) On both compartment, the pressure was generally decreased throughout the range of motion. Linear correlation on both compartment showed statistically significant throughout the range on motion, with higher correlation value on the lateral compartment. Total 66 additional ligament balancing procedures were performed.

**Conclusion:**

Using orthosensor, we could obtain 94% quantified balance knee, consequently. And between the techniques, measured resection TKA showed less balanced knee and also required more additional procedures compared to modified gap balancing TKA. Furthermore, with the acquired quantified data during appropriate ligament balancing, the surgeon could eventually reduce the complications associated with soft tissue imbalance in the future.

## Background

A successful outcome in total knee arthroplasty depends on various surgical factors including precise bone resection, correct alignment of the components, femoral and tibial rotation, and above all adequate soft tissue balancing [1–3]. Technological advances over the past few years have facilitated accurate alignment and rotation in the surgical field via three-dimensionally guided bone resection such as navigation or ROBODOC systems [4, 5]. However, adequate soft tissue balancing still remains a challenge for many surgeons, especially younger surgeons who lack surgical experience. Further, experienced surgeons traditionally obtain soft tissue balance using their own subjective “feeling” rather than a scientific perspective [6–9]. The soft tissue balancing “feeling” is affected by factors such as surgical experience, patient’s BMI, gender, generalized laxity, degree of joint contracture and even the surgeon’s daily condition [10, 11].

Despite the excellent long-term clinical results of TKA, with reported survival greater than 95% at 15 years [12, 13], a few patients experience failure. Many factors contributing to dysfunction and pain have been reported [14]. Especially, revision arthroplasty due to instability has been estimated in more than 20% each year, which may be due to inappropriate ligament balancing [15]. Soft tissue balancing of the knee during total knee arthroplasty is a significant factor for postoperative patient satisfaction and implant longevity [16]. Unfortunately, until recently, soft tissue balancing, which can lead to catastrophic failure of the implant, has been determined only by surgeon’s judgement with subjective feeling.

**Fig. 1:**
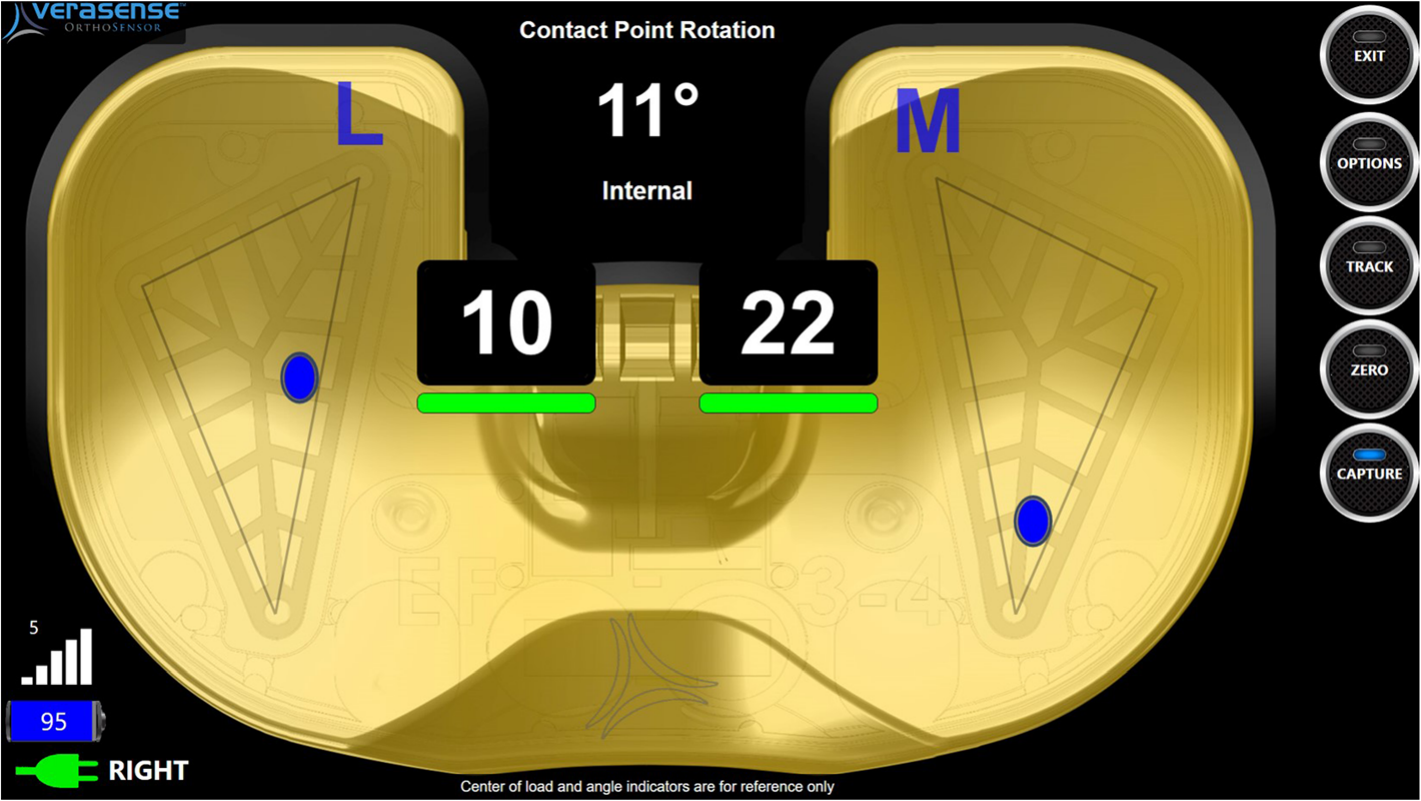
Quantification of medial and lateral compartment pressure using VERASENSE

Combining the need for objective soft tissue balancing and advanced technology, VERASENSE Knee System (OrthoSensor Inc., Dania Beach, FL) has been introduced, recently. VERASENSE is an orthosensor that enables surgeons to quantify ligament balance based on real-time, evidence-based data during primary and revision TKA (Fig. 1). This disposable device delivers wireless data to an intra-operative monitor to facilitate informed decision-making regarding implant position and soft-tissue releases to improve balance and stability through a full range of motion.

The purpose of this study was: (1) to quantify the compartment pressure of the joint throughout the range of motion during TKA using orthosensor, (2) to determine the usefulness of orthosensor by analyzing correlation between the pressure in both compartment with initial trial and after final implantation, and (3) to evaluate the types and effectiveness of additional ligament balancing procedures to compartment pressure.

## Methods

### Patient demographic data

Between July 2017 and March 2018, 84 patients (84 knees) who showed varus deformed osteoarthritic knees underwent unilateral posterior cruciate ligament-retaining (CR) or -sacrificing (PS) primary total knee arthroplasty using VERASENSE Knee System (OrthoSensor Inc., Dania Beach, FL) in our hospital. The surgical procedure was approved by our institutional review board (IRB) of Chonnam National University Hwasun Hospital, and written informed consent was obtained from all patients. All patients were implanted with the same total knee system (NexGen™, Zimmer, Inc., Warsaw, IN, USA). Bone resection was performed by either measured resection technique using ROBODOC® system or modified gap balancing technique. Among 84 knees, measured resection TKA was performed in 34 patients (17 CR implants and 17 PS implants) and modified gap balancing TKA was performed in 50 patients (all PS implants). Patients were consisted of 11 men and 73 women ranging in age from 51 to 83 years (mean age: 69.6 years old) at the time of surgery. There were 46 left knees and 38 right knees. The mean mechanical axis was 10.5° varus (range 0.5 to 26.7°). The CR implant was used in 17 patients with mean 9.2° varus deformity (range 0.7 to 19.5°) and the PS implant was used in 67 patients with mean 10.8° varus deformity (range 0.5 to 26.7°). Patients younger than 50 years, those who underwent revision TKA, or previous ligamentous reconstruction, osteotomy, or any other procedures, were excluded from the study.

Preoperatively, all patients underwent standard anteroposterior, lateral radiographic examination, Merchant & Lauren’s view, Rosenberg view and standing extremity teleoroentgenography for evaluation of mechanical alignment. Further, we obtained the preoperative valgus and varus stress views to evaluate the ligament laxity of the knee preoperatively, and helical computed tomography (CT) to prepare ROBODOC® planning for measured resection TKA. All the surgical procedures were carried out by a senior author (EKS) using either ROBODOC® (Curexo Technology Corp, Fremont, CA, USA)-assisted TKA or conventional TKA using modified gap balancing technique.

### Surgical procedure and VERASENSE application

#### Bone resection (measure resection and modified gap balance technique)

All knees were exposed with a standard midline incision with medial parapatellar arthrotomy, and the patella was everted laterally. In ROBODOC®-assisted TKA, the patient’s leg was rigidly connected to the robot and bony landmarks of both femur and tibia were registered using a probe. After the registration was successfully accomplished, bone resection was automatically conducted by ROBODOC® according to preoperative planning using ORTHODOC system with measured resection technique. In the coronal plane, distal femur and proximal tibia cutting were used to cut perpendicular to the mechanical axis, with 7° of posterior slope to the mechanical axis of the tibia in the sagittal plane. Femoral rotational alignment was planned perpendicular to the trans-epicondylar axis and the tibial rotational axis was planned parallel to that of the femur [17]. In conventional TKA, after exposing the knee joint, bone resection was performed by tibia first modified gap balance technique. Tibial preparation was carried out using extramedullary cutting guide with proximal tibial resection perpendicular to the mechanical axis with 7° posterior slope. Tibial component rotation was aligned to the line connecting the posterior cruciate ligament insertion site and the medial edge to medial 1/3 of the tibial tuberosity. Distal femoral preparation was carried out by intramedullary rod guide with 5° of valgus, and femoral rotation was determined according to the balanced flexion gap [18].

#### Intraoperative compartment pressure evaluation using VERASENSE

**Fig. 2:**
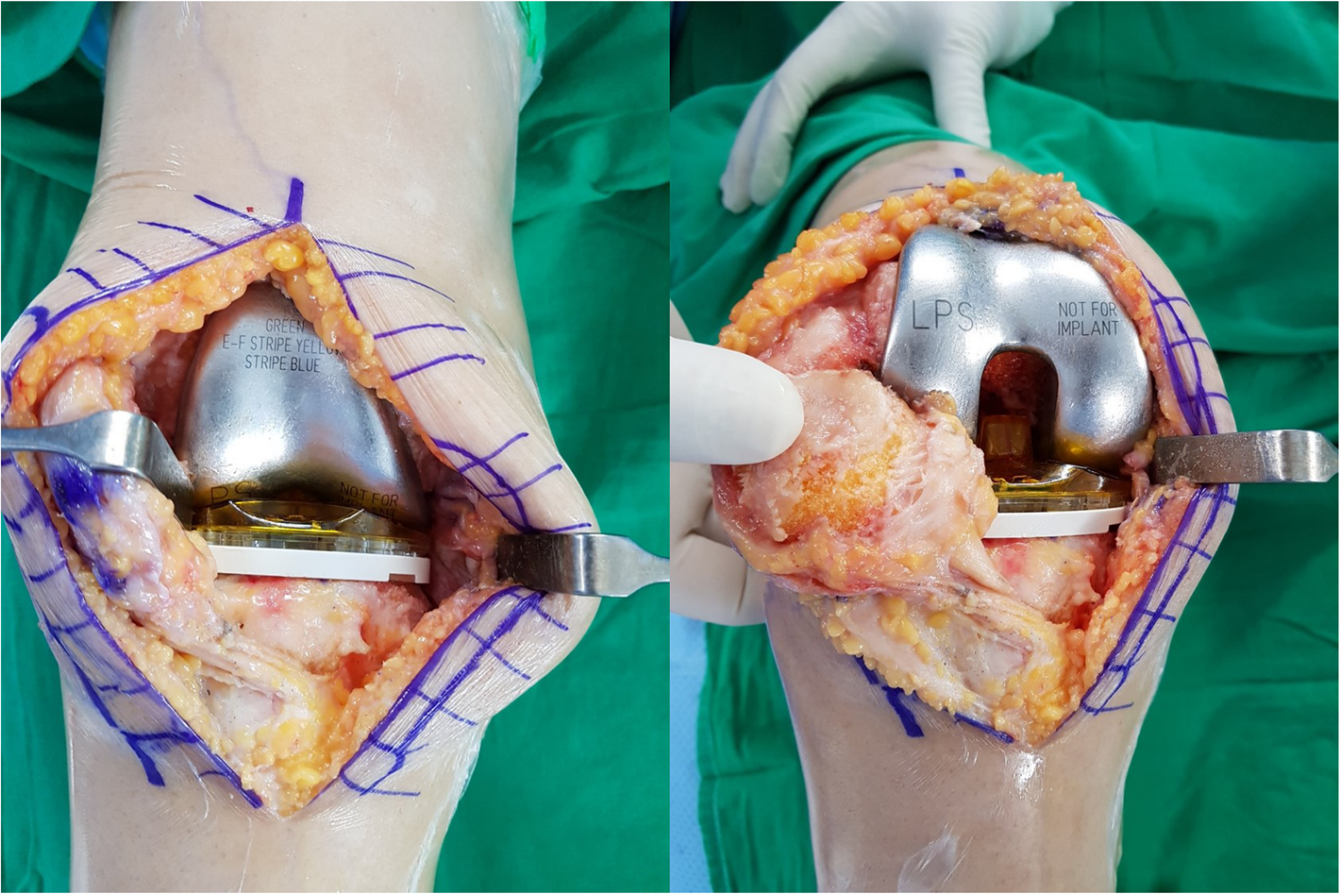
VERASENSE inserted in the tibial tray in extension and flexion

The VERASENSE Knee System (OrthoSensor Inc., Dania Beach, FL) is a wireless and disposable articular loading quantification device, which is inserted in the tibial component tray during the surgery. The sensor allows quantification of the intraoperative real-time loading data in both medial and lateral compartments of the knee via the full range of movement (Fig. 2).

Once the bony resection was carried out either with the ROBODOC®-assisted measured resection or modified gap balance technique, initially femoral and tibial trial components were implanted and the VERASENSE sensor was inserted into the tibial component tray for initial (INI) quantitative assessment of compartment pressure. After reducing the patella and closing the capsule with one or two towel clips, a gentle range of motion was performed by the surgeon. The surgeon held the leg in a neutral position and monitored the medial and lateral loading forces from full extension to full flexion. In order to observe additional details, we divided the range of motion into 5 categories and recorded compartment pressure in: full extension, 30°, 60°, 90° and full flexion. Less than differential loading of 15 pounds between the medial and lateral compartments, was considered as adequately “balanced” according to previous studies [19]. After initial ligament balance assessment, if the joint showed imbalance (loading difference > 15 pounds), additional soft tissue releases (s) or bony resection were performed and recorded. After each additional ligament balancing procedure, the VERASENSE was inserted in the tibial tray and compartment pressure was re-measured. The orthosensor was routinely re-zeroed before the insertion to minimize the error due to plastic deformation of the sensor. Resultingly, the compartment pressures were recorded initially right after the bone resection (INI), after each additional procedure, and after the final implantation of the real femoral and tibial components with the bone cement (FIN).

#### Additional ligament balancing procedures

In “unbalanced” knees, various ligament balancing procedures were performed. For soft tissue balancing procedures, we generally used “pie-crusting” (PC) technique with an 18-guage needle while releasing the ligaments [20]. Medial collateral ligament (MCL) release using the PC technique was most frequently used in soft tissue releases, followed by sub-periosteal superficial MCL release using a narrow curved chisel, lateral collateral ligament (LCL) release using PC technique, and iliotibial band (ITB) release. When the joint was too tight to balance by soft-tissue release alone or in valgus-aligned knee after the initial procedure, additional bony resection was performed. Varus or additional proximal tibial recut was mostly performed followed by varus or additional distal femur recut, additional osteophyte removal on medial compartment and posterior tibial slope resection.

#### Postoperative radiologic evaluation

For radiologic evaluation, standard anteroposterior, lateral and standing extremity teleoreontgenography were used to evaluate the postoperative alignment. Mechanical axis, femorotibial angle (FTA), and posterior slope of the tibia (PTS) were measured from the postoperative radiograph. Also to evaluate the adequate positioning of the implants, coronal and sagittal inclination of femoral and tibial components (α, β, γ, δ) were measured using anteroposterior and lateral radiographs. To determine the intra-observer variation, the radiographic measurement was repeated by the author (observer A) after 1 week. To determine the inter-observer variation, the measurements were performed by 3 experts: the author (observer A), another knee orthopedic surgeon (observer B), and a radiologist (observer C).

### Statistical analysis

Means, standard deviations and frequencies were calculated, and paired t-tests were used to evaluate the continuous variables and chi-square test was used for categorical variables. Multiple regression analyses were made to evaluate the intraoperative effectiveness of additional procedures on compartment pressure. Linear correlation analysis was performed to evaluate the relationship between the initial and final pressure differences in both compartments. For radiographic measurement, intra-observer consistency between the two sets of measurements obtained by observer A and inter-observer consistency between the three sets of measurements obtained by observers A, B, and C, were analyzed using Pearson’s correlation coefficient and the intra-class correlation coefficient (ICC). ICC > 0.75 was regarded as excellent, ICC 0.40–0.75 was fair to good, and ICC < 0.40 was poor (20). A *P* value less than 0.05 was considered statistically significant and all statistical analyses were performed using SPSS 24.0 (SPSS, Chicago, IL). Finally, a power analysis was conducted to estimate the required number of the study. The power was set at 80% or higher with *p* < 0.05, and at least 64 knees were required for the study.

## Results

**Fig. 3:**
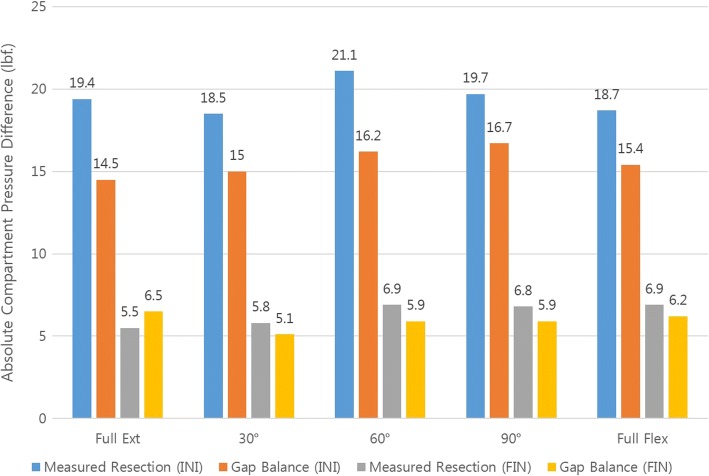
Initial (INI) and final (FIN) absolute mediolateral pressure difference in measured resection (M) and modified gap balance techniques (G)

**Fig. 4:**
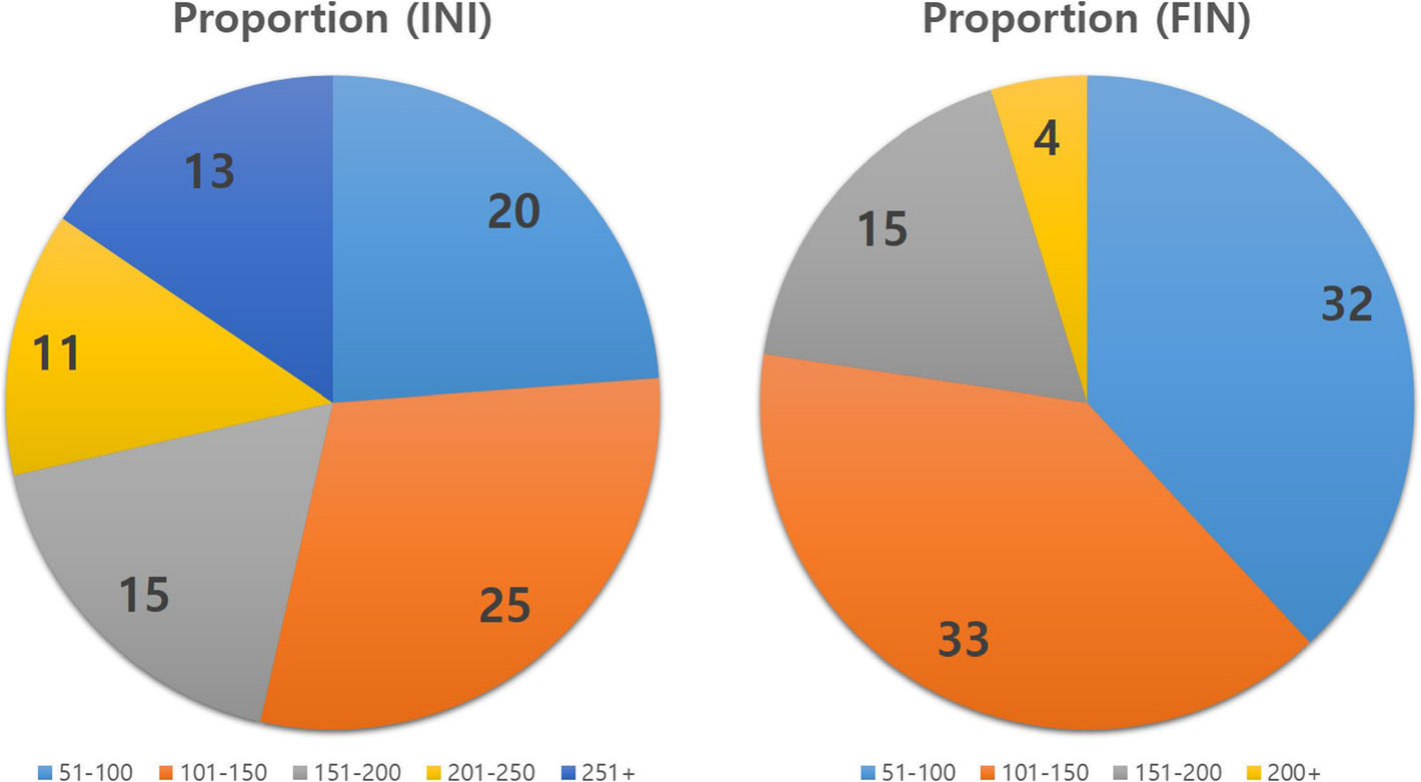
Proportion of knees according to initial (INI) and final (FIN) compartment pressure

Absolute mediolateral compartment pressure difference determined initially (INI) and finally (FIN) were recorded and are shown in Fig. 3. Throughout the range of motion, the measured resection group showed higher initial pressure difference than the modified gap balancing group. Upon initial trial and final measurement after implantation, the proportion of the knees according to the compartment pressure (sum of medial and lateral compartment pressure) is shown in Fig. 4. After final implantation, 95% of the patients showed total compartment pressure less than 200 lbf.

**Fig. 5:**
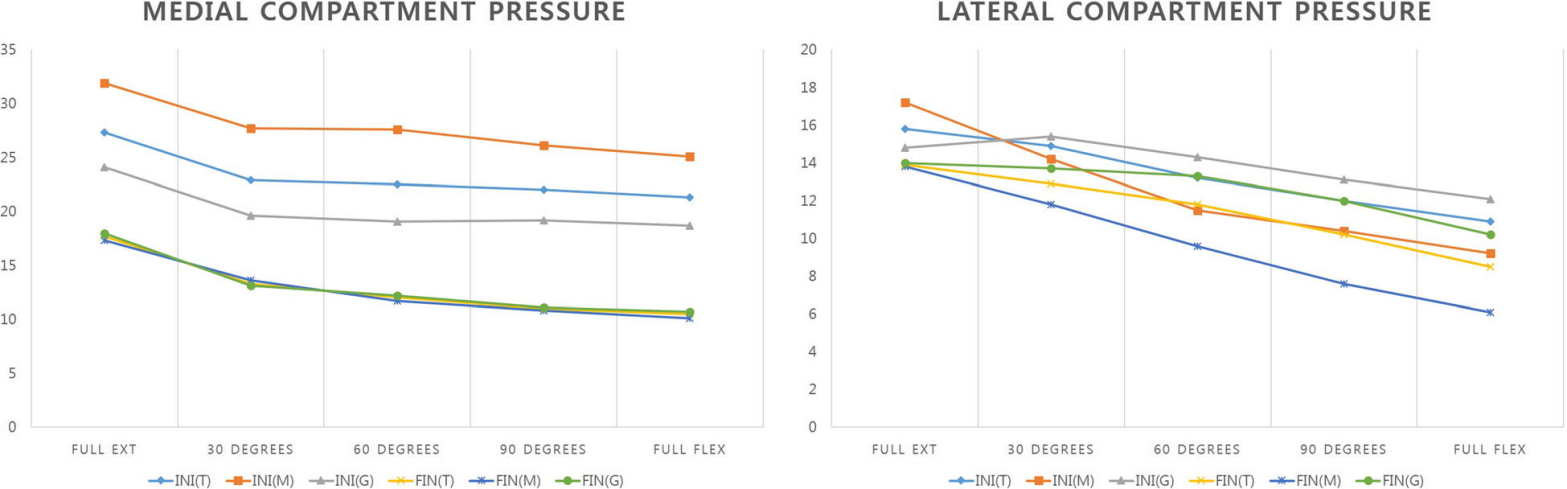
Initial (INI) and final (FIN) average compartment pressure of overall (T), measured resection (M) and modified gap balance technique (G)

Overall (T), measured resection TKAs (M) and modified gap balancing TKAs (G) initial (INI) and final (FIN) compartment pressure on medial and lateral side are shown in Fig. 5. In both medial lateral compartments, the pressure was generally decreased throughout the ROM in both TKA methods. In the medial compartment, the overall final compartment pressure (FIN) was significantly decreased compared with initial pressure (INI) throughout all range of motion (*p* < 0.05), and in the lateral compartment, the overall final compartment pressure was also decreased throughout the ROM especially during 30° flexion and full flexion (*p* < 0.05) (Table 1). According to TKA methods, both TKA techniques showed significant decrease in the medial compartment at the final measurement, although the lateral compartment did not show significant decrease in both technique (Table 2).

**Table 1:** Overall Comparison of Initial (INI) and Final (FIN) Compartment Pressure

	Initial (lbf.)	Final (lbf.)	*P*-value^a^
Medial compartment			
Full Extension	27.3 ± 25.8	17.7 ± 7.8	0.001
30°	22.9 ± 25.0	13.3 ± 5.6	0.000
60°	22.5 ± 24.2	12.0 ± 6.3	0.000
90°	22.0 ± 25.7	11.0 ± 6.8	0.000
Full Flexion	21.3 ± 21.5	10.5 ± 7.9	0.000
Lateral compartment			
Full Extension	15.8 ± 11.7	13.9 ± 6.3	0.082
30°	14.9 ± 11.3	12.9 ± 5.6	0.046
60°	13.2 ± 11.3	11.8 ± 6.0	0.174
90°	12.0 ± 11.4	10.2 ± 6.5	0.074
Full Flexion	10.9 ± 11.7	8.5 ± 6.6	0.017

^a^Paired-t test. The *p* values of < 0.05 was considered significant

**Table 2:** Initial (INI) and Final (FIN) Compartment Pressure Classified by TKA technique: Measured Resection (M) vs. Modified Gap Balance (G) technique

	Initial (lbf.)	Final (lbf.)	*P*-value
Medial (G)			
Full Extension	24.1 ± 18.7	18.0 ± 8.6	0.023
30°	19.6 ± 19.0	13.1 ± 5.1	0.025
60°	19.1 ± 18.4	12.2 ± 5.6	0.009
90°	19.2 ± 20.8	11.1 ± 5.9	0.006
Full Flexion	18.7 ± 19.5	10.7 ± 7.5	0.002
Lateral (G)			
Full Extension	14.8 ± 9.8	14.0 ± 6.7	0.510
30°	15.4 ± 11.1	13.6 ± 5.8	0.190
60°	14.3 ± 11.3	13.3 ± 6.2	0.400
90°	13.1 ± 11.9	12.0 ± 9.6	0.365
Full Flexion	12.1 ± 12.6	10.2 ± 6.6	0.129
Medial (M)			
Full Extension	31.9 ± 33.5	17.3 ± 6.3	0.009
30°	27.7 ± 31.5	13.6 ± 6.2	0.008
60°	27.6 ± 30.4	11.7 ± 7.2	0.003
90°	26.1 ± 31.3	10.8 ± 8.1	0.006
Full Flexion	25.1 ± 30.4	10.1 ± 8.6	0.005
Lateral (M)			
Full Extension	17.2 ± 14.0	13.8 ± 5.9	0.075
30°	14.2 ± 11.8	11.8 ± 5.2	0.129
60°	11.5 ± 11.3	9.6 ± 5.0	0.286
90°	10.4 ± 10.5	7.6 ± 5.3	0.105
Full Flexion	9.2 ± 10.2	6.1 ± 5.8	0.061

^a^Paired-t test. The p values of < 0.05 was considered significant

^b^*M* Measured resection, ^c^*G* Modified gap balance

**Fig. 6:**
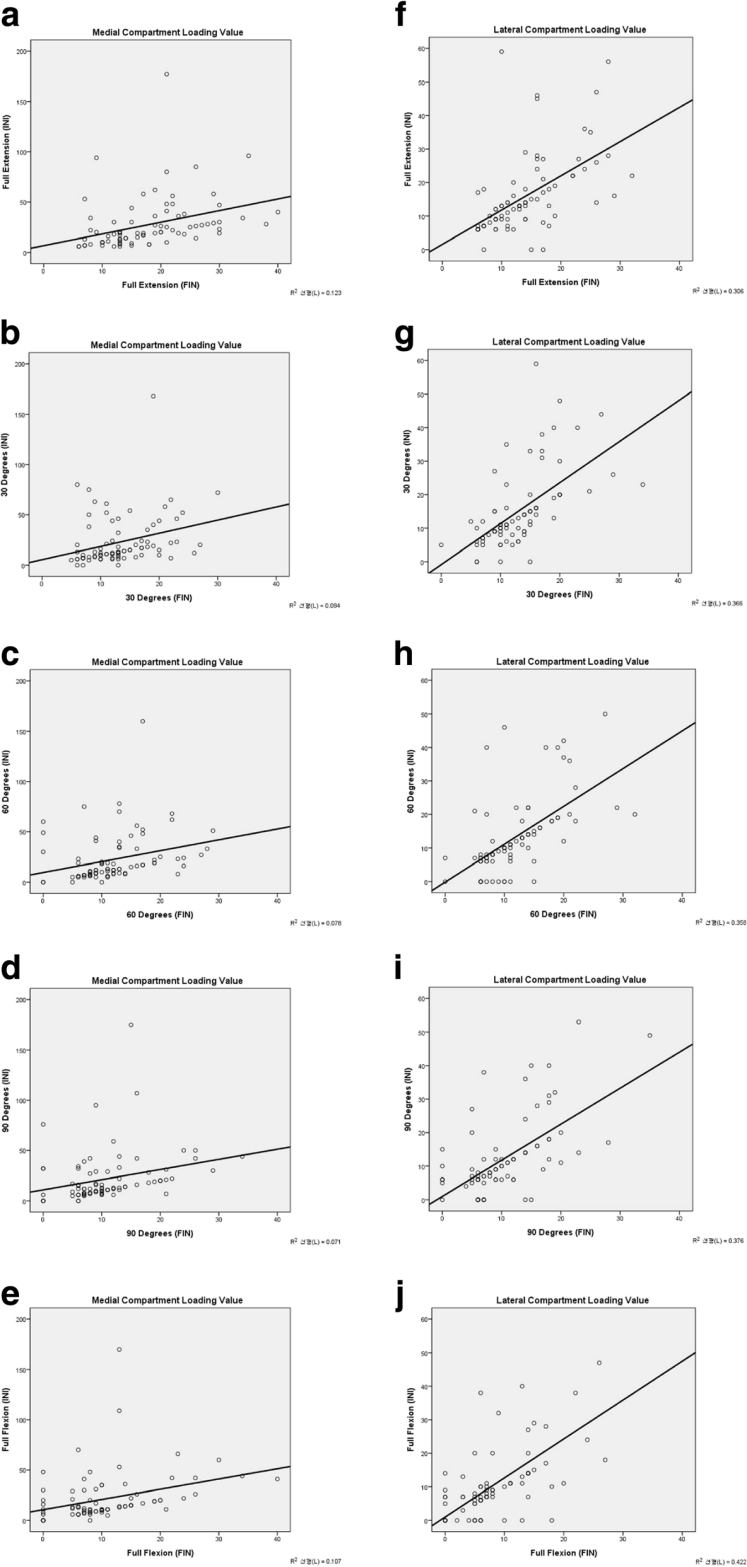
**a**-**j** Linear correlation between initial (INI) and final (FIN) compartment pressure

Linear correlation in the medial and lateral compartments was both statistically significant throughout the range of motion, with higher correlation in the lateral compartment. Medial compartment coefficients of determination were as follows: full extension (R^2^ = 0.12; *P* = 0.01), 30° flexion (R^2^ = 0.084; *P* = 0.007), 60° flexion (R^2^ = 0.078; *P* = 0.010), 90° flexion (R^2^ = 0.071; *P* = 0.014), and full flexion (R^2^ = 0.107; *P* = 0.002). Lateral compartment coefficients of determination were as follows: full extension (R^2^ = 0.306; *P* = 0.000), 30° flexion (R^2^ = 0.366; P = 0.000), 60° flexion (R^2^ = 0.358; P = 0.000), 90° flexion (R^2^ = 0.376; P = 0.000), full flexion (R^2^ = 0.422; P = 0.000) (Fig. 6).

Thirty patients (35.7%) showed “balanced” knee without a need for additional ligament balancing upon initial measurement and 54 patients showed “imbalance” knee who underwent additional procedures. Among 30 balanced knees, 6 were measured resection TKAs and 24 were modified gap balancing TKAs. After initial bone resection, modified gap balancing TKAs showed significantly higher proportion of “balanced” knees than measured resection TKAs (24 of 50 TKAs vs. 6 of 34 TKAs) (*P* = 0.004). Upon final pressure measurement after ligament balancing and component implantation, 79 patients (94.0%) showed a “balanced” joint with medial and lateral pressure difference less than 15 pounds throughout the full range of motion. Compared with the initial “balanced” knee (35.7%), there was significant improvement following ligament balancing at the final implantation (94.0%) (*P* < 0.05).

For 54 imbalanced knee, total 66 additional ligament balancing procedures (including soft tissue release (s) and bony resection) were carried out: 38 procedures for 28 measured resection TKAs and 28 procedures for 26 modified gap-balancing TKAs. Nine distal femur recuts (varus and neutral resection), 14 proximal tibia recuts (varus and neutral resection), 4 osteophyte removals, and 1 tibia posterior slope recut constituted additional bony procedures; and 26 medical collateral ligament (MCL) releases using pie-crusting (PC) method, 5 sub-periosteal superficial MCL releases, 5 iliotibial band (ITB) releases using PC method, 2 lateral collateral ligament (LCL) releases using PC method were performed for additional soft tissue releases.

The change of compartment pressure due to each additional procedure has been evaluated individually throughout the range of motion, and statistically significant procedures analyzed by regression analysis at each motion, are shown in Table 3. Results of regression analysis of the additional procedures were calculated based on the contributing beta values for each procedure by subtracting the post-procedure compartment pressure from pre-procedure compartment pressure. A positive value indicates improvement in joint pressure, and negative value indicates increased joint pressure while the absolute value indicates effectiveness of the joint pressure improvement. The effect of LCL release using PC method and tibia posterior slope resection procedures could not be analyzed due to lack of cases. However, the loading has been calculated to identify the effect of additional procedures on the compartment pressure throughout the range of motion. On average, bony resection showed relatively higher beta values compared with soft tissue releases. Distal femur recut showed a statistically significant effect on full extension and 90° flexion; proximal tibia recut showed significant effect on full extension; MCL release with PC technique showed effect throughout the range of motion but only significant in full extension; and release of suferficial MCL showed small but significant effect throughout the full range of motion.

**Table 3:** Regression analysis of Additional Ligament Balancing Procedures on the joint

	Full Extension	30°	60°	90°	Full Flexion
Distal femur recut	*23.8* ^a^	43.1	40.7	*19.1* ^a^	16.8
Proximal tibia recut	*15.0* ^a^	19.6	42.0	42.6	29.2
Osteophyte removal	12.1	−6.4	4.8	3.6	7.6
MCL^b^ PC^c^	*16.6* ^a^	27.0	32.9	34.0	34.0
Sub-periosteal superficial MCL release	*9.8* ^a^	*3.1* ^a^	*7.6* ^a^	*− 2.6* ^a^	*7.2* ^a^
ITB^d^	22.7	−9.6	15.2	*2.6* ^a^	*2.5* ^a^

^a^Multiple regression analysis. The *p* values of < 0.05 was considered significant

^b^MCL Medial collateral ligament, ^c^PC Pie-crusting technique, ^d^ITB Iliotibial band

On postoperative radiograph, average mechanical axis (MA) was 1.8° varus with 4.7° valgus femorotibial angle (FTA) and 6.2° posterior tibial slope (PTS). According to implant radiologic measurements, femoral and tibial components were also adequate positioned overall (Table 4). And the radiologic measurements showed excellent intra-observer consistency and inter-observer consistency across the 3 observers (Table 5).

**Table 4:** Postoperative Radiologic Measurement of the Implant

	α (°)	β (°)	γ (°)	δ (°)
Average	95.2	89.5	2.8	86.4
SD^t^	2.6	2.2	1.9	2.3

α Coronal medial inclination of femoral component, β Coronal medial inclination of tibial component, γ Sagittal inclination of femoral component, δ Sagittal inclination of tibial component, ^t^SD Standard Deviation

**Table 5:** Comparison of intra-observer and inter-observer consistency in Radiologic measurements

Observer	A-A		A-B		A-C		B-C	
	ICC^¥^	*P*-value^a^	ICC	*P*-value	ICC	*P*-value	ICC	*P*-value
MA^b^	0.934	0.005	0.862	0.014	0.871	0.009	0.864	0.015
FTA^c^	0.944	0.004	0.877	0.008	0.854	0.012	0.869	0.010
PTS^d^	0.962	0.005	0.812	0.018	0.828	0.022	0.836	0.015
α	0.952	0.002	0.834	0.012	0.852	0.011	0.847	0.007
β	0.961	0.001	0.882	0.010	0.904	0.005	0.891	0.009
γ	0.918	0.010	0.807	0.024	0.833	0.019	0.829	0.022
δ	0.933	0.005	0.856	0.010	0.872	0.009	0.886	0.005

^a^Pearson’s-correlation test. The p values of < 0.05 was considered significant

^b^MA Mechanical Axis, ^c^FTA FemoroTibial Angle, ^d^PTS Posterior Tibial Slope, α Coronal medial inclination of femoral component, β Coronal medial inclination of tibial component, γ Sagittal inclination of femoral component, δ Sagittal inclination of tibial component, ^¥^ICC Intra-class Correlation Coefficient

## Discussion

The most important implication of the study is that, not only measured resection TKA but also modified gap balancing TKA using subjective “feeling” of ligament balancing, can be inaccurate or can show variable results despite abundant experience. Objective quantification using real-time orthosensor improved the soft tissue balance in both TKA techniques.

Until now, most of the studies about kinematics of knee joint have been based on biomechanical models or cadaveric studies [21–23]. Unfortunately, these studies have limitations in understanding the dynamics of the real knee joint. It is difficult to extrapolate the findings from cadaveric studies to living human body due to bias associated with factors such as postmortem contracture and tissue atrophy. Furthermore, in the biomechanical model studies, data application was a demanding procedure, because data are generally acquired from limited specimens.

Further, according to previous studies, although TKAs were carried out in conventional methods with soft tissue balancing intraoperatively, the potential imbalance remains due to subjective ligament balancing. It resulted in a variety of postoperative complications such as instability, stiffness, loosening, etc., which resulted in a significant proportion of TKA failures and revision surgeries [24, 25].

Therefore, we used the VERASENSE system to ensure adequate ligament balancing objectively via quantification of the joint intraoperatively, which would reduce the postoperative complications in long-term follow-up.

The initial (INI) and final (FIN) absolute pressure difference between medial and lateral compartments was higher in measured resection TKAs compared with gap balancing technique TKAs. As the femur resection was conducted according to the soft tissue balance in the modified gap balance technique, the quantification results were obtained as expected. But the difference was relatively higher than expected in spite of modified gap technique bone resection. Theoretically, modified gap balance bone resection should not show pressure difference between medial and lateral compartments because the bone resection was already based on patient’s ligament balance. In previous studies, the superiority of measured resection and gap balancing technique was still disputed. A few studies reported better outcomes with the gap balancing technique TKAs, whereas other reports showed no significant difference between the techniques in long-term clinical outcomes [3, 26, 27].

The overall initial (INI) and final (FIN) average compartment pressure showed a significant decrease throughout the range of motion in the medial compartment (*p* < 0.05). In the lateral compartment, the overall final loading values were significantly decreased under 30° flexion and full flexion (*p* < 0.05). Correlation analysis suggested that both medial and lateral compartments showed a significant relationship between initial (INI) and final (FIN) pressure throughout the range of motion. Our study indicated that variability of pressure between the initial (INI) and final (FIN) measurements was equal in both compartments, which facilitated surgical prediction of similar loading measurements with both VERASENSE system and final implanted components. Consequently, the surgeon could expect the same measured compartment pressure with the final implanted components.

Another important implication of the study was that only 30 patients (35.7%), constituting only 1/3 of the patients showed initially “balanced” knee after measured resection or gap balancing TKA. In spite of the abundant experience of the surgeon (EKS) in total knee arthroplasty for more than 30 years, subjective human “feeling” is variable and inaccurate. Fortunately, after additional ligament balancing procedures, a total of 79 patients (94.0%) completed the “balanced” TKA procedure, with load difference lower than 15 lbs. In a previous report of TKAs with a load difference lower than 15 lbs., better shorter-term clinical outcomes were observed compared with “unbalanced” knees with a difference greater than 15 lbs. [19], which underscored the significance of the objective quantification orthosensor, for adequate and satisfactory results. As mentioned before, the subjective and inaccurate procedures associated with traditional ligament balancing [28] have been elucidated through the study.

Excluding the 30 patients who were initially “balanced”, 54 patients underwent additional ligament balancing procedure. A total of 66 additional procedures were performed, which accounted for an average of 1.2 procedures per patient. According to our study, generally measured resection TKA patients underwent more additional ligament balancing procedures than modified gap balance TKA patients, 1.36 per person and 1.1 per person, respectively. Bony resection showed higher changes in joint loading, especially distal femur recut was effective in full extension to mid-flexion, and proximal tibia recut was effective during the mid-flexion. These results were similar to previous qualitative studies. For example, Mihalko et al. [29] demonstrated that distal femur resection was effective for mid-flexion contractures. Ahn et al. [30] demonstrated the effectiveness of proximal tibia varus resection on severe varus deformities. Among soft tissue release (s), MCL release with PC technique was most frequently used and was also most effective through flexion, which also was similar to many previous studies using MCL release as an important procedure during total knee arthroplasty in varus deformity [31–33]. Although superficial MCL has been known to have a lesser effect in flexion stability [34], our study showed that sub-periosteal superficial MCL release showed small but significant effect on joint loading throughout the range of motion. Postoperative radiographic alignment of patients including mechanical axis, femorotibial angle, posterior slope, and implant positions were within satisfactory range.

However, this study also has a few limitations. First, the group only consisted of 84 patients, and a larger group may have yielded more generalized analyses of the knee joint. Fortunately, our study findings were similar to those reported previously in published studies. Second, we used only a single implant system in all patients. Other implant systems showed different loading values due to differences in implant design. Also, the PCL release being a strong influence on flexion ligament balancing, using two types of implant (CR and PS type) may have produced some bias during interpretation of the data. Third, although the pressure was quantified and presented as digited results on the screen, the measurement was done by a single surgeon, suggesting possible bias. Fourth, we did not compare the long-term clinical and radiological outcomes between objectively balanced TKAs and traditionally subjectively balanced TKAs.

## Conclusion

By objective quantification using orthosensor, we observed significant decrease in both medial and lateral compartments pressure after TKA, and could obtain 94% balanced knee, consequently. And between the techniques, measured resection TKA showed less balanced knee in the initial pressure measurement and also required more additional procedures compared to modified gap balancing TKA. But also, we suggest that regardless of TKA surgical methods, additional procedures could be needed for adequate “patient-specific” ligament balancing. Furthermore, with the consistent data of the orthosensor acquired during appropriate ligament balancing, the surgeon could eventually reduce the complications associated with soft tissue imbalance in the future.

## Abbreviations

CR: Cruciate-retaining
CT: Computed tomography
FIN: Final
FTA: Femorotibial angle
ICC: Intra-class correlation coefficient
INI: Initial
ITB: Iliotibial band
LCL: Lateral collateral ligament
ma: Mechanical axis
MCL: Medical collateral ligament
PC: Pie-crusting technique
PS: Posterior-sacrificing
PTS: Posterior tibial slope
TKA: Total knee arthroplasty
